# Association between wasting and food insecurity among children under five years: findings from Nepal demographic health survey 2016

**DOI:** 10.1186/s12889-020-09146-x

**Published:** 2020-06-29

**Authors:** Sajama Nepali, Padam Simkhada, Ian Glynn Davies

**Affiliations:** 1 Masters in Food and Nutrition, Central Department of Home Science, Tribhuvan University, Kathmandu, Nepal; 2 Associate Dean International and Professor of Global Health, School of Human and Health Sciences, University of Huddersfield, Huddersfield, UK; 3 Reader in Nutritional Science, School of Sport and Exercise Sciences, Liverpool John Moores University, Liverpool, UK

**Keywords:** Association, Children, Food insecurity, Intervention, Socio-demographic, Wasting

## Abstract

**Background:**

Wasting is a consequence of food insecurity, inappropriate dietary practices, and inadequate caring and feeding practices. The present study assessed association between wasting and household food insecurity among under 5 years old children, along with other socio-demographic characteristics.

**Methods:**

This study is a secondary analysis of the Nepal Demographic and Health Survey 2016. The survey is cross-sectional in design with use of standardized tools. The sampling frame used is an updated version of the frame from the 2011 National Population and Housing Census. The participants were children under 5 years of age (*n* = 2414). Logistic regression was carried out to identify the odds of being wasted for children belonging to different levels of food insecure households using odds ratio and 95% confidence intervals.

**Results:**

The prevalence of wasting increased with the level of food insecurity, from mild (9.4%) to moderate (10.8%) and to severe (11.3%). The highest proportions of wasted children were in Province 2 (14.3%), from rural areas (10.1%), born to mothers with no education (12.4%) and from a richer quintile (11.3%). Children belonging to severe food insecure households had 1.36 (95%CI 0.72–2.57) adjusted odds of being wasted and those belonging to mild food insecure and moderately food insecure households had 0.98 (95%CI 0.64-1.49) and 1.13 (95%CI 0.65–1.97) odds of being wasted respectively. Province 1 (AOR 2.06, 95%CI 1.01–4.19) and Province 2 (AOR 2.45, 95%CI 1.22–4.95) were significantly associated with wasting.

**Conclusion:**

Considering the increment in childhood wasting as per level of food insecurity, an integrated intervention should be developed in Nepal that, 1. addresses improving knowledge and behavior of community people with respect to diet and nutrition; 2. reduce the problem of food insecurity through agricultural interventions.

## Background

Wasting is a severe phenomenon of losing weight and is often associated with acute starvation and severe diseases [1]. It is a consequence of food insecurity, poor access to appropriate diet, inadequate caring and feeding practices such as exclusive breastfeeding or low quantity and quality of complementary food, unaffordable health care, lack of a sanitary environment including access to safe water, sanitation and hygiene services [2]. Children suffering from wasting are susceptible to long term developmental delays, vulnerable to several infections and face an increased risk of death, particularly when wasting is severe [3].

Wasting has affected 7.5% or 50.5 million children of under 5 years worldwide, of which 16 million are severely wasted [3]. More than half of all wasted children lived in South Asia in 2018, demanding a serious need of help with appropriate and adequate interventions [4]. Furthermore, wasting contributes to 4.7% of all deaths among children aged under 5 years globally and severe wasting is associated with 2 million deaths per year, as severely wasted children are 11 times more likely to die compared to healthy children [5]. The mortality rate elevates when a child is both stunted and wasted [5]. The prevalence of wasting among children under 5 years was 10% in 2016 [6], which was reduced from 11% in 2011 as per Nepal Demographic Health Survey (NDHS) [7].

Globally, 26.4% of the world population or 2 billion people suffer from moderate and severe food insecurity [8]. In Nepal, 52% of households are suffering from food insecurity and do not have adequate access to food throughout the year [6]. Of which, mild food insecurity accounts for 20% and moderate and severe food insecurity holds 22% and 10% respectively. This high prevalence of food insecurity in the nation could be one of the underlying factors for the high occurrence of different forms of undernutrition such as stunting, wasting and underweight [8]. Nepal is behind other countries in securing food security, as the trend of food insecurity inclined by only 1 % from 2011 to 2016 [6]. However, Nepal has committed to achieve Sustainable Development Goal 2 to bring hunger to a level of zero, [9] and international organizations such as the World Food Program is building up government capacity to reach the hunger goal to zero [10].

Subsequently, less studies have been performed in Nepal investigating the relationship between wasting and food insecurity and no evidence is available for recognizing the risk factors of childhood wasting and understanding the extent to which risk factors contributes to wasting. In this respect, the objective of this study was to present the distribution of wasting according to various levels of food insecurity as well as socio-demographic characteristics and to measure the risk of wasting as determined by those circumstances to help policy makers and program planners to develop the health interventions adequately and appropriately as per the scientific evidence provided.

## Methods

### Data

The freely available data from NDHS 2016 was used for this study and secondary analysis was conducted with the study population as children under 5 years. NDHS is a nationally representative cross sectional survey conducted every 5 years [11]. The NDHS dataset was used due to availability of the nature of data required for this research and considering the large sample size NDHS incorporates, representing national population that warrants a high precision of the findings. Alongside, reliable and standardized tools were used allowing for comparison between countries.

### Sampling

The sampling frame used for the NDHS 2016 is an updated version of the frame from the 2011 National Population and Housing Census, conducted by the Central Bureau of Statistics. Following March 2017, urban and rural areas were termed as “Nagarpalika” and “Gaonpalika” respectively after the structural changes forming 263 urban municipalities or Nagarpalikas that resulted into 59% of the total population living in urban areas. The results of 2016 NDHS were built on the updated urban-rural classification and the sample was stratified and selected in two stages in rural areas and three stages in urban areas. In rural areas, wards were selected as primary sampling units (PSU), and households were selected from the sample PSUs. In urban areas, wards were selected as PSUs, one Enumeration Areas (EA) was selected from each PSU, and then households were selected from the sample EAs [6]. The detail of sampling is provided elsewhere [6]. Only, the children of interviewed women were measured for their anthropometric characteristics [12]. The total number of children under 5 years analyzed in this study was 2414.

### Variables

#### Wasting

The outcome variable was wasting. It was defined as the percentage of children aged 0 to 59 months, whose weight for height is below minus two standard deviations (moderate) and minus three standard deviations (severe wasting) from the median of the World Health Organization Child Growth Standards [1].

#### Food insecurity

The main explanatory variable was food insecurity. Food security is defined as “a situation that exists when all people, at all times, have physical, social and economic access to sufficient, safe and nutritious food that meets their dietary needs and food preferences for an active and healthy life” [13]. The questionnaire for food insecurity was adopted from the United States Agency for International Development’s Food and Nutrition Technical Assistance project [6]. The questions captured household perceptions of food vulnerability or stress and behavioral responses to food insecurity and were arranged in order of severity and frequency of occurrence. Based on the responses, four food insecurity categories were created; i) food secure households, ii) mildly food insecure households, iii) moderately food insecure households, and iv) severely food insecure households. The detail questionnaire is included elsewhere [6].

#### Socio-demographic variables

The socio-demographic variables included in this study were wealth quintiles, place of residence, mother’s education, province and sex of child based on conceptual framework of undernutrition and literature review [14, 15]. Wealth quintile was recorded as (i) poorest (ii) poorer (iii) medium (iv) richer and (v) richest. The wealth quintile was based on a household’s ownership of selected assets, such as televisions and bicycles; materials used for housing construction; and types of water access and sanitation [16]. Place of residence was categorized into (i) rural and (ii) urban. Mother’s education level was categorized into (i) no education (ii) primary (iii) secondary (iv) higher. Child sex was recorded as male and female and province was categorized as per the seven provinces of Nepal.

#### Tools

Demographic Health Survey (DHS) core questionnaires were adapted by NDHS and contextualized for Nepalese population. Questionnaires were finalized after the pre-test to ensure reliability and validity of the tools [6]. Face-to-face interviews were performed for data collection.

### Statistical analysis

The 23rd version of Statistical Package for Social Science (IBM USA) was used for data analysis. Descriptive statistics identified the frequency of wasting. Logistic regression identified the odds of being wasted for children belonging to different levels of food insecure households taking into account other independent variables using odds ratio and 95% confidence intervals (CI). *P* value < 0.05 was considered statistically significant for the association between wasting and independent variables. The sample-weighted data was taken into consideration during analysis. The complex sample analysis method was used to account for the study design and sample weight [17].

### Ethics

Nepal Health Research Council and human research ethics committee in ICF Macro International approved NDHS 2016 [6]. Approval was granted from Independent Review Boards of New Era and ICF Macro International for all the data collection tools and procedures for NDHS. Written informed consent were taken from respondents. The dataset was requested for access from DHS program website [6].

## Results

### Distribution of wasted children under 5 years by household food insecurity and socio-demographic characteristics

Table 1 shows the frequency and percentage of wasted and non-wasted children below 5 years of age by household food insecurity, wealth quintiles, sex of child, mother’s education, place of residence and provinces. The prevalence of wasting in children below 5 years of age increased with the level of food insecurity from mild (9.4%) to moderate (10.8%) and to severe food insecurity (11.3%). Out of urban children, 9.2% were wasted in urban and 10.1% were wasted in rural areas. The highest proportions of wasted children were in Province 2 (14.3%), born to a mother with no education (12.4%) and were from a richer quintile (11.3%). Additionally, chi-square tests showed significant associations between wasting and mother’s education (*P* = 0.014) and province (*P* < 0.001).

**Table 1:** Frequency and percentage for wasted and non-wasted children under 5 years by household food insecurity and socio-demographic characteristics

Characteristics	Wasted (< − 2 SD)		Non-wasted (≥ − 2 SD)	
	N	%	N	%
**Household food Insecurity (*N*** **= 2414,*P*** **= 0.496)**				
Secure	88	8.9	900	91.1
Mild	76	9.4	733	90.6
Moderate	41	10.8	339	89.2
Severe	27	11.3	211	88.7
**Wealth quintiles (*N*** **= 2412,*P*** **= 0.296)**				
Poorest	43	8.7	449	91.3
Poorer	48	9.1	478	90.9
Middle	58	10.6	491	89.4
Richer	59	11.3	465	88.7
Richest	23	7.2	298	92.8
**Sex of child (*N*** **= 2414,*P*** **= 0.837)**				
Male	119	9.5	1134	90.5
Female	113	9.7	1048	90.3
**Mother’s education (*N*** **= 2360, P = 0.014)**				
No education	102	12.4	723	87.6
Primary	42	8.9	429	91.1
Secondary	64	8.6	684	91.4
Higher	23	7.3	293	92.7
**Place of residence (*N*** **= 2414,*P*** **= 0.408)**				
Urban	117	9.2	1161	90.8
Rural	115	10.1	1021	89.9
**Province (*N*** **= 2414,*P*** **< 0.001)**				
Province 1	46	11.8	344	88.2
Province 2	95	14.3	570	85.7
Province 3	15	4.2	339	95.8
Province 4	11	5.9	176	94.1
Province 5	34	7.5	418	92.5
Province 6	11	7.1	144	92.9
Province 7	20	9.5	191	90.5

Children who slept in the household the night before the survey were selected for analysis. Data weighted according to DHS recommendations [17]. *P* value < 0.05 was considered statistical significant for chi square test between wasting and explanatory variables

### Association between wasting and household food insecurity and socio-demographic characteristics

Table 2 shows the crude odds ratio (COR) and adjusted odds ratio (AOR) with 95% CI for wasting and its relationship with household food insecurity, wealth quintiles, sex of child, mother’s education, place of residence and provinces. Children belonging to severe food insecure households had 1.36 (95% CI 0.72–2.56) adjusted odds of being wasted and those belonging to mild food insecure and moderately food insecure households had 0.98 (95%CI 0.64-1.49) and 1.13 (95%CI 0.65–1.97) odds of being wasted respectively compared to those belonging to food secure households. Children born to mothers without any education had 1.84 (95% CI 1.08–3.12) crude odds of being wasted than those born to mothers with higher education; however, no significant association was seen in the adjusted analysis (AOR-1.35, 95% CI 0.71–2.59). Province 1 (AOR-2.06, 95%CI 1.01–4.19) and Province 2 (AOR-2.45, 95%CI 1.22–4.95) were significantly associated with wasting.

**Table 2:** COR and AOR with 95% CI between wasting and household food insecurity and socio-demographic characteristics

Variables	COR	95% CI		AOR	95% CI	
		Lower	Upper		Lower	Upper
**Household food Insecurity (*N*** **= 2414)**						
Mild	1.08	0.75	1.54	0.98	0.64	1.49
Moderate	1.24	0.75	2.06	1.13	0.65	1.97
Severe	1.38	0.80	2.38	1.36	0.72	2.57
Secure	1			1		
**Wealth quintile (*N*** **= 2412)**						
Poorest	1.226	0.68	2.22	1.00	0.42	2.34
Poorer	1.316	0.72	2.40	0.87	0.41	1.83
Middle	1.513	0.88	2.60	0.86	0.45	1.66
Richer	1.625	0.89	2.95	1.15	0.60	2.23
Richest	1			1		
**Sex of child (*N*** **= 2414)**						
Female	1.03	0.75	1.42	1.05	0.75	1.46
Male	1			1		
**Mother’s education (*N*** **= 2360)**						
No education	1.84	1.08	3.12	1.35	0.71	2.59
Primary	1.27	0.68	2.37	1.04	0.52	2.07
Secondary	1.21	0.68	2.16	1.07	0.57	2.03
Higher	1			1		
**Place of residence (*N*** **= 2414)**						
Rural	1.13	0.82	1.56	0.99	0.72	1.36
Urban	1			1		
**Province (*N*** **= 2414)**						
Province 1	2.18	1.06	4.50	2.06	1.01	4.19
Province 2	2.74	1.46	5.14	2.45	1.22	4.95
Province 3	0.70	0.30	1.64	0.64	0.27	1.53
Province 4	1.34	0.67	2.69	1.24	0.61	2.49
Province 5	1.32	0.63	2.76	1.15	0.55	2.40
Province 6	1.66	0.82	3.37	1.54	0.75	3.15
Province 7	1			1		

Children who slept in the household the night before the survey were selected for analysis. Data weighted according to DHS recommendations [17]. Complex sample analysis method was used to account for the study design and sample weight

## Discussion

The objective of this study was to present the distribution of wasting according to socio-demographic characteristics with a focus on household food insecurity and to measure the risk of wasting as determined by those characteristics. In this study, the prevalence of wasting among children under 5 years increased with the levels of food insecurity from mild (9.4%) to moderate (10.8%) and to severe (11.3%) food insecurity steadily, which is in accordance with the study of John et al. reporting a dose-response relation between severity of food insecurity and children’s nutritional health [18]. Another study in low and middle income countries such as Bangladesh and Vietnam found that moderate and severe food insecurities were significantly associated with wasting [19]. However, no significant association was found in the present study. This was surprising given the established association of food insecurity and children’s nutritional status by the United Nations Children Fund conceptual framework of undernutrition. It may have been due to the small sample size between wasted children and children belonging to food insecure households. A larger study is recommended to further understand this relationship in Nepal. Furthermore, confounding variables may influence wasting such as knowledge of mothers on nutrition, health seeking behaviors, maternal nutritional status, access to health services, and quality of environmental conditions such as maintenance of hygiene and availability of sanitation facilities. Attention should be towards reducing food insecurities in order to combat the problem of wasting considering more than half of the population of Nepal are suffering from food insecurity, particularly focusing on rural areas and poor people where the severity is high. Government of Nepal has made several attempts to improve food security. Right to Food and Food Sovereignty Act 2018 was declared as a fundamental right as per the 18.3 constituents of interim constitution of Nepal [20]. This act demands all citizens have the right to food and right to food security, and aims to protect people from the negative effects of increasing food insecurity, uneven distribution of food and lack of access to food [21]. Further to this, the act supports the Sustainable Development Goal 2 to bring hunger to a level of zero. Besides, in collaboration with the United States Agency for International Development, the 14 most vulnerable districts in the west and 11 districts in the east have been supported for improving food security through livelihood interventions and building resilience to mitigate impacts of climate change [9]. Similarly, other organizations such as the World Food Program is strengthening the capacity of government in improving food security, adequate nutrition, and emergency preparedness and response, in order to support the country to achieve zero hunger and to graduate from a low income country to a lower middle-income country by 2023 [10]. Apart from interventions addressing food insecurity, integrated interventions are implemented in the nation after release of the Multi-sector Nutrition Plan I and II that addresses education, sanitation, feeding behavior of sub-groups in population such as adolescent girls, pregnant and lactating women and their infants among the rural and poorer and poorest groups [22]. However, the high prevalence of wasting and food insecurity is still a challenge to the nation that needs urgent attention.

The thematic report on food security and nutrition showed greater prevalence of wasting in female (36.1%) than male children (33.8%), which contradicts the findings of the present study showing similar prevalence of wasting among male (9.5%) and female (9.7%) children. Harding et al. reported that sex was not significantly associated with wasting in Nepal aligning with the present study [23]. However, they also noted that the association was noted significant in other low income countries such as Afghanistan, India, Bangladesh, and Pakistan [23].

The prevalence of wasting among the wealth quintiles was not uniform. The lowest prevalence was noted in the richest quintile and highest prevalence was noted in a richer quintile in this study. The second lowest prevalence was observed among the poorest quintiles. There was no significant association between wasting and wealth quintile. A multinational cohort study conducted in Ethiopia, India, Peru and Vietnam found children belonging to the lowest quintile households had significantly increased probabilities of being wasted in all four countries in comparison to children belonging to the highest quintile households [24]. This is not consistent with the result of the present study having noted the higher prevalence of wasting among the middle and richer quintiles.

The highest prevalence of wasting was noted in Province 2, which might be due to a large volume of underprivileged people living in Province 2 lacking basic facilities such as education and health [25]. Additionally, the Nepal Multidimensional Poverty Index 2018 reports more than 47.9% of people living in Province 2 are multi-dimensionally poor, which is greater by almost 20% than that of the national average (28.6%) [26].

The Demographic and Health Surveys of 15 sub-Saharan African countries showed that urban–rural differentials are considerable in all countries, that they have narrowed in most countries primarily due to an increase in urban undernutrition [27]. Another Nepalese study showed a significantly higher prevalence of wasting was found in rural areas as compared to urban areas [28]. Aligning with these studies, the prevalence of wasting in the present study was slightly higher among children living in rural (10.1%) than those living in urban areas (9.2%); however, the difference is small. The superior availability of health and sanitation facilities and good education among people living in urban areas on taking care of children might contribute to the low prevalence of wasting in urban areas than the rural areas [29].

The highest proportion of wasted children were born to mothers without education (12.4%), which is similar to the finding of a thematic report on nutrition and food security stating that the highest proportion (43.3%) of undernourished children were born to mothers without education [28]. Asfaw et al. in the Southern region of Ethiopia showed a significant association between mother’s education and all three indicators of undernutrition (stunting, wasting and underweight) [30] again in agreement with the present study. Educated mothers have a good knowledge on child care, are likely to take care of sanitation and follow hygiene adequately, helping to improve the nutritional status of their children [31].

Due to the cross-sectional nature of the study, the causal inference between wasting and study variables could not be estimated. However, this study has given provincial level information on wasting, which is not available in other studies of Nepal and is highly beneficial for formulating provincial level policies. The provincial level information on wasting would bring the focus on the current need of each province and this information would support the design of interventions within various provinces. The study warrants a high precision of the findings due to a large sample size representing the Nepalese national population. Reliable and comparable standardized tools were utilized.

## Conclusion

The prevalence of wasting increased with the level of food insecurity from mild to moderate and to severe. Considering this increment, interventions addressing wasting should consider addressing the problem of food insecurity along with improving the knowledge and behavior of community people with respect to diet and nutrition in Nepal. The governmental and non-governmental interventions should target the food insecure households to reduce the incidence of child wasting. Socio-demographic aspects of wasting should be incorporated into nutritional interventions to reduce risk of wasting in vulnerable populations. Lastly, policies that promote availability, access, and consumption of diverse nutrient rich foods need to be encouraged, especially for those vulnerable groups where such foods have the potential to reduce the impacts of food insecurity.

## Data Availability

The data that support the findings of this study are available from the DHS website but restrictions apply to the availability of these data, which were used under license for the current study, and so are not publicly available. Data are however available upon reasonable request.

## Abbreviations

AOR: Adjusted Odds Ratio
CI: Confidence Intervals
COR: Crude Odds Ratio
DHS: Demographic Health Survey
EA: Enumeration Areas
NDHS: Nepal Demographic Health Survey
PSU: Primary Sampling Unit
